# Resting-State Functional Connectivity Differences in College Students with and without Food Insecurity

**DOI:** 10.3390/nu14102064

**Published:** 2022-05-14

**Authors:** Nicolas Guerithault, Samuel M. McClure, Chinedum O. Ojinnaka, B. Blair Braden, Meg Bruening

**Affiliations:** 1 College of Health Solutions, Arizona State University, Phoenix, AZ 85004, USA; guerithaultnicolas@gmail.com (N.G.); chinedum.ojinnaka@asu.edu (C.O.O.); 2 Department of Psychology, Arizona State University, Tempe, AZ 85287, USA; samuel.mcclure@asu.edu

**Keywords:** food insecurity, executive function, college students, functional connectivity, fMRI, resting-state, cognitive function, frontoparietal network, default-mode network, salience network

## Abstract

We used functional magnetic resonance imaging (fMRI) to investigate cross-sectional differences in functional connectivity across cognitive networks at rest among age and sex matched college students with very low food security [food insecurity (FI); *n* = 20] and with high food security (*n* = 20). The participants completed the Behavior Rating Inventory of Executive Function-2 (BRIEF-2) and Adverse Childhood Experiences (ACEs) questionnaires. Seven-minute resting-state fMRI scans were collected. Independent Component Analysis assessed group connectivity differences in three large-scale networks: the default-mode network (DMN), the frontoparietal network (FPN), and the salience network (SN). FI was associated with poorer Global BRIEF scores (adjusted β = 8.36; 95% CI: 2.32, 14.40) and five BRIEF subscales: Inhibit, Initiate, Working Memory, Plan, and Organize (*p*-values < 0.05). The students with FI had greater functional connectivity between the FPN and left middle temporal gyrus (cluster size p-FWE = 0.029), the SN and precuneus (cluster size p-FWE < 0.001), and the SN and right middle frontal gyrus (cluster size p-FWE = 0.016) compared to the students with high food security. Exploratory correlations revealed that greater connectivity between the SN and right middle frontal gyrus was associated with poorer BRIEF Inhibit scores (*p* = 0.038), and greater connectivity between the FPN and left middle temporal gyrus was associated with poorer BRIEF Organize scores (*p* = 0.024) for the students with FI. Greater functional connectivity between the FPN, DMN, and SN at rest may contribute to executive function difficulties for college students with FI.

## 1. Introduction

The most recent report from the United States Department of Agriculture indicates that 38 million Americans live in households that are food insecure, which is defined as having inconsistent access to adequate and healthy foods [1]. Food insecurity is related to a poorer quality diet [2] and is linked to a greater risk of obesity, eating disorders [3], cardiometabolic diseases [4], stress, depression [5], anxiety, and sleep disorders [6]. Rates of food insecurity are disproportionately high among populations of color and households with children, especially those led by single parents [1]. There are also high rates of food insecurity among college students [7,8], and food insecurity among college students is associated with poorer academic performance and a lower grade point average [9,10].

Food insecurity has been linked to objectively poorer cognitive performance across the lifespan [11,12], although the neurological underpinnings of this relationship remain unknown. Studies of food insecurity and cognitive function have primarily focused on middle-aged and older populations; no studies have tested for differences in college students, despite their high rates of food insecurity. It has been shown among college students that poorer executive functioning, a set of higher order cognitive processes involved in planning and regulating behavior, is associated with worse academic outcomes [13,14]. Studies have also found that measures of social cognition, which involves regulating behaviors that are socially learned, are predictive of eating behavior and physical activity [15,16]. Both adults and children with food insecurity exhibit objectively poorer executive functioning [17,18,19,20]. There is a need to determine whether college students with food insecurity similarly experience deficits in executive functions, given the implications for academic success and health behaviors. Furthermore, our recent systematic review found that no published studies of food insecurity have ever incorporated functional brain measures in their approach [11]. This is a critical gap in the literature, and a clearer understanding of the neural correlates of food insecurity will help further our understanding of the link between cognition and food insecurity.

Cognitive processes are known to depend upon synchronous activity across distinct brain regions [21]. Functional magnetic resonance imaging (fMRI) can be used to study connectivity between brain regions while individuals lay awake at rest (resting-state fMRI), which reveals predictable patterns of network activity [22]. A wide range of cognitive disorders and deficits are now linked to connectivity differences across three major large-scale networks: the default-mode network (DMN), the frontoparietal network (FPN), and the salience network (SN) [23]. In a healthy cognitive state, the DMN and FPN tend to be anti-correlated with one another; the former increasing its activity during undirected and self-referential thought [24], and the latter becoming active through goal-directed thought or executive control [25]. The SN plays a role in shifting between these two cognitive and brain states as appropriate [26,27,28]. Given that these three intrinsic networks are especially important for higher-order cognition [23], we investigated for differences in resting-state connectivity involving these networks, and explored whether these differences related to self-reported measures of executive function.

## 2. Materials and Methods

### 2.1. Recruitment of Subjects

This cross-sectional, exploratory study recruited forty individuals from the Phoenix metropolitan area who were enrolled full time as undergraduate students at Arizona State University. As a pilot study, sample size was determined based on convenience. Recruitment and data collection occurred between February and March of 2020 (prior to the university shutdown of human subjects research due to COVID-19) and from September 2020 until April 2021 (after human subjects research was allowed to be restarted). To compare students with high and very low food security, participants were age and sex matched (20 males and 20 females) so that there were twenty matched pairs of food secure (*n* = 20) and food insecure (*n* = 20) participants in total. All interested participants completed an online screening questionnaire to assess eligibility for the study. The screening questionnaire remained open until we completed brain scans of each of the 20 matched pairs. The questionnaire assessed food insecurity status, disordered eating behaviors, and other exclusion factors. Individuals were excluded if they were parents, pregnant, left-handed, non-native English speakers, not 18–25 years old, or reporting disordered eating or marginal or low food security (see below). The Eating Disorder Examination Questionnaire [EDE-Q] [26] was administered during screening. Individuals reporting scores greater than one were excluded due to the possible confounding effects of disordered eating behavior on brain function [27]. In total, 3399 people took the screening survey; of those, 147 were eligible for participation based on inclusion and exclusion criteria. However, not all eligible participants were contacted for participation because there may not have been an age or sex match for them. The Arizona State University Institutional Review Board approved all study protocols (study number: 00010783).

### 2.2. Survey Measures

At the time of neuroimaging, we again collected measures of food insecurity and socio-demographics, as well as measures of executive function and adverse childhood experiences (ACEs).

*Food security.* Food security status was determined using the 10-item Adult Food Security Survey Module [28], assessing experiences with food insecurity over the past 12 months with four distinct categories: 0 = High food security, 1–2 = Marginal food security, 3–5 = Low food security, and 6–10 = Very low food security. Given the known issues with assessing food insecurity among college students [8], only participants with high food security (score = 0) or very low food security (score = 6–10) were included in the study. As a pilot study, comparing the two extremes of food security and low food security allowed was done to maximize the effect sizes expected for brain and cognitive differences across the groups. However, this approach did not allow for continuous assessment of how marginal and low food security is related to our outcome measures.

*Executive function.* Executive function was assessed using the self-report Behavior Rating Inventory of Executive Function-2 Adult Version (BRIEF-2A) [29]. This validated questionnaire assesses a Global Executive score and nine distinct domains: Inhibit, Self-Monitor, Plan/Organize, Shift, Initiate, Task Monitor, Emotional Control, Working Memory, and Organization of Materials. In this 75-item measure, the participants were asked to respond to the frequency (never, sometimes, often) in which they experienced difficulties concentrating on tasks or trouble accepting different ways to solve problems with work, friends, or tasks. Higher BRIEF scores are associated with poorer executive function across all subscales.

*ACEs.* The CDC ACEs Questionnaire [30] was administered to all participants at the time of the MRI. Adverse experiences during childhood have been linked to greater odds of food insecurity [31] and poorer cognitive function among young adult populations [32]. ACEs were summed and used as a continuous variable.

*Socio-demographics.* We also collected self-report data on sex assigned at birth, age, race/ethnicity, and Pell grant status (yes/no). Race and ethnicity were measured with the US Census Bureau items. In total, there were four Hispanic White participants, seven non-Hispanic and non-White participants, and five Hispanic participants of other races. Given the limited sample, for the purposes of analyses race/ethnicity was classified as white and other.

### 2.3. fMRI Data Acquisition

Structural and functional magnetic resonance imaging (MRI) data were collected using a 3.0 Tesla GE scanner at the Banner Alzheimer Institute in Phoenix, AZ, USA. High-resolution T1-weighted gradient images were acquired sagittally using a BRAVO pulse sequence (interleaved, bottom-up acquisition; 0.9 mm slice thickness, field of view = 230 mm). Resting-state functional blood-oxygen level dependent (BOLD) gradient echo-planar images were then acquired (sequential, bottom-up acquisition; 3 mm slice thickness, field of view = 200 mm, repetition time = 2000 ms, echo time = 30 ms, flip angle = 84°). Padding was used to minimize head movement during the scans, and participants received earplugs and headphones to protect against the scanner noise. The resting-state scans were collected over 7 min, and participants laid with eyes open looking at a fixed crosshair on the screen.

### 2.4. Survey Analyses

Given that the sample was sex and age matched, bivariate analyses were limited to the association between food insecurity and ACEs (*t*-test) and race/ethnicity (chi-squared test). A set of ten separate multivariable linear regression models assessed relationships between food insecurity and each of the nine BRIEF subscales and the Global Executive score, adjusting for ACEs. Analyses were conducted in Stata Statistical Software: Release 15 (College Station, TX, USA: StataCorp LP).

### 2.5. fMRI Preprocessing and Analysis

Preprocessing of resting-state images was performed in Matlab (Mathworks, Natick, MA, USA) using Statistical Parametric Mapping (SPM-12; Wellcome Department of Cognitive Neurology, Institute of Neurology, London, UK). Prior to co-registration, T1 images were skull-stripped and segmented into grey matter, white matter, and cerebrospinal fluid. Functional images underwent slice-timing correction to correct for timing differences in slice acquisition, and realignment was performed to correct for head movement. The images were then co-registered to the skull-stripped T1 images, normalized to Montreal Neurological Institute space using the Dartel method [33], and spatially smoothed using an 8 mm full-width half-maximum Gaussian kernel. Scrubbing parameters were generated using the Artifact Detection Toolbox to capture scans where relative displacement exceeded 2 mm, and both scrubbing and realignment motion timeseries were entered as 1st-level covariates in the analysis.

The CONN Toolbox (https://www.nitrc.org/projects/conn, accessed on 1 June 2021) was used to analyze group differences in resting-state functional connectivity. A group independent component analysis (ICA) data-driven approach was used to parse forty distinct sources of temporally-coherent signals within the subjects’ data. Forty components were chosen as this was the default number in CONN Toolbox [34]. Each component was spatially matched with a series of canonical networks. The best spatial matches for the DMN, SN, and FPN were visually inspected and subsequently considered in the analysis. Group differences in whole-brain functional connectivity between each of the three network components and cluster regions were assessed using a family-wise-error (FWE)-corrected cluster size threshold of α < 0.05. ACE scores were entered into CONN toolbox as a covariate for the connectivity analyses. Connectivity values were then extracted for each subject. Correlations with BRIEF subscale scores that showed group differences were assessed to determine if functional connectivity could be a neural mechanism for executive function differences associated with food insecurity. The correlation analyses were exploratory, thus an α < 0.05 with no corrections for multiple comparisons was implemented.

## 3. Results

### 3.1. Demographics and Self-Reported Measures

The bivariate analyses indicated a significantly greater frequency of ACEs among the students with food insecurity compared to those with food security (Table 1). There were no significant group differences in food insecurity based on race/ethnicity. After adjusting for ACEs, the linear models indicated an association between food insecurity and the Global Executive BRIEF score, as well as five BRIEF subscales: Inhibit, Initiate, Working Memory, Plan, and Organize (*p*-value’s < 0.05; Table 2) where significantly higher BRIEF scores (poorer cognitive function) were observed among the students with food insecurity as compared to the food-secure students. These associations were related to weak to moderate Cohen’s d effect sizes (Table 2).

### 3.2. Resting-State Functional Connectivity Group Differences and Correlations with BRIEF

**Figure 1:**
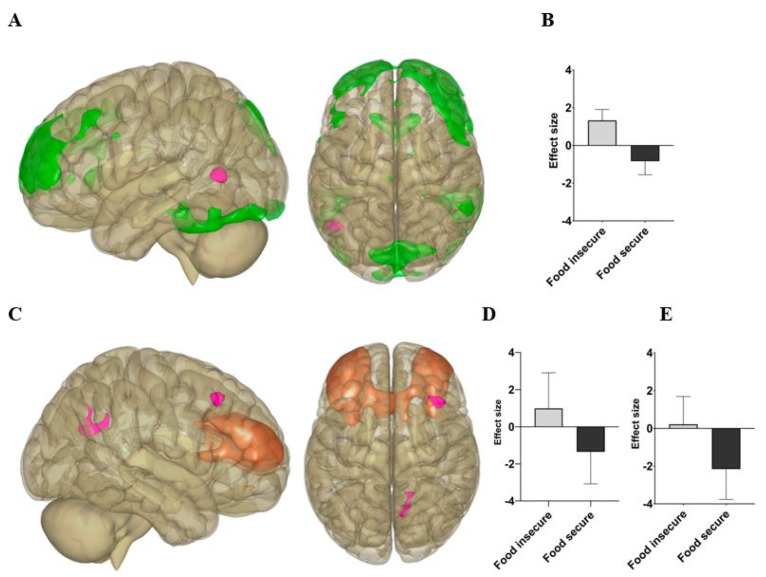
(**A**) Left and superior views of the Frontoparietal Network (FPN) component (green) and a cluster within the left middle temporal gyrus (pink). (**B**) Group differences in connectivity between the FPN and left middle temporal gyrus. (**C**) Right and superior views of the Salient Network (SN) component (orange) and two clusters (pink) within the precuneus posteriorly and right middle frontal gyrus anteriorly. (**D**) Group differences in connectivity between the SN and precuneus. (**E**) Group differences in connectivity between the SN and right middle frontal gyrus.

There were significant group differences in resting-state functional connectivity involving the FPN and SN components but not the DMN component, after adjusting for ACEs (Table 3). In whole-brain analyses, those with food insecurity showed significantly greater connectivity between the FPN and left middle temporal gyrus (Figure 1; Table 3: p-Family-wise error (FWE) = 0.029), between the SN and precuneus (Figure 1; Table 3: p-FWE < 0.001), and between the SN and right middle frontal gyrus (Figure 1; Table 3: p-FWE = 0.016).

**Figure 2:**
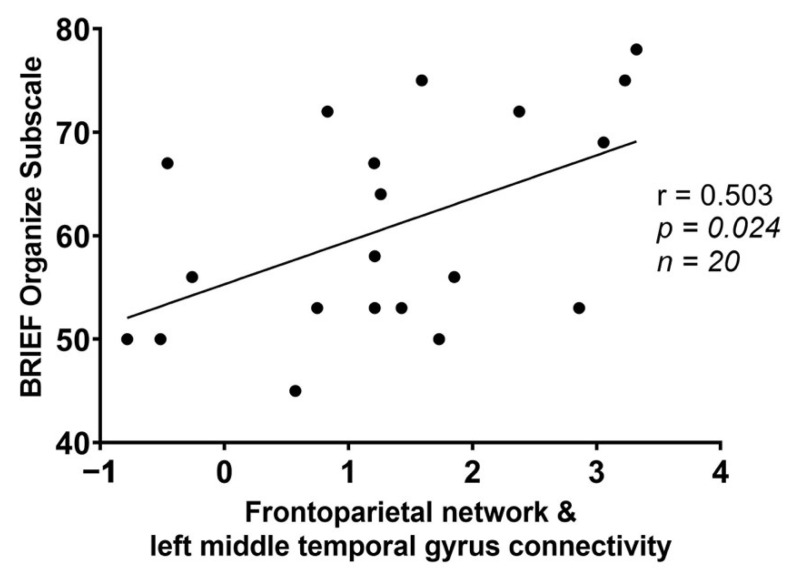
Scatterplot of Behavior Rating Inventory of Executive Function (BRIEF) Organize scores and connectivity between the frontoparietal network and a cluster within the left middle temporal gyrus in the food insecure group (r = 0.503; *p* = 0.024).

**Figure 3:**
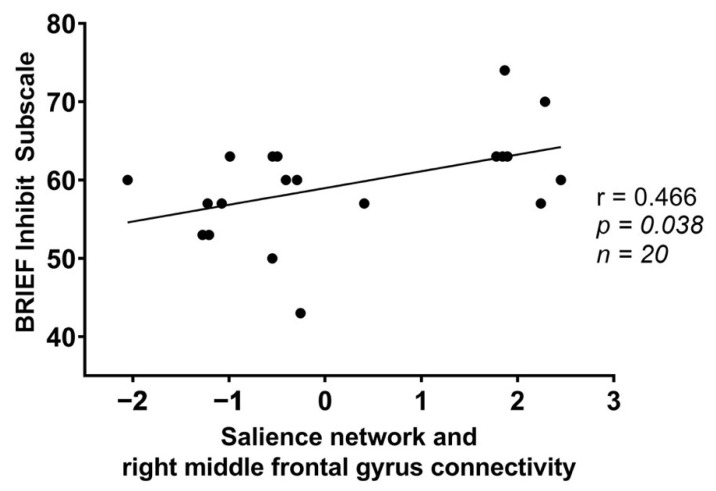
Scatterplot of Behavior Rating Inventory of Executive Function (BRIEF) Inhibit scores and connectivity between the salience network and a cluster within the right middle frontal gyrus in the food insecure group (r = 0.466; *p* = 0.038).

Within the food insecure group, significant correlations were found between FPN and left middle temporal gyrus connectivity and the BRIEF Organize subscale (Table 4: *p* = 0.024), and between the SN and right middle frontal gyrus and the BRIEF Inhibit subscale (Table 4: *p* = 0.038). Exploratory correlations indicated greater FPN and left middle temporal gyrus connectivity among the food insecure group was associated with higher BRIEF Organize scores (Figure 2). Greater SN and right middle frontal gyrus connectivity among the food insecure group was associated with higher BRIEF Inhibit scores (Figure 3).

## 4. Discussion

The purpose of this study was to explore neural mechanisms of cognitive differences associated with food insecurity in college students using resting-state fMRI. To our knowledge, this is the first functional neuroimaging study of individuals experiencing food insecurity, and the first to show how brain function is affected in college students with food insecurity. Our findings indicate that students with food insecurity have poorer executive function and differences in functional connectivity between key cognitive networks compared to students with high food security.

Food insecurity has previously been associated with impaired executive function in both children and adults [17,18,19,20]; however, research examining the relationship between food insecurity and executive function is lacking for college students. In our study, we found that very low food security was associated with significantly poorer global BRIEF scores. One future goal for this work should be to identify in what specific way executive function is impacted by food insecurity. The BRIEF measure allowed us to conduct exploratory analyses to test for differences in nine executive functioning subscales. We found lower scores on five of the nine subscales after adjustment for ACEs: Organize, Plan, Working Memory, Initiate, and Inhibit.

College students with food insecurity tend to have lower grade point averages [35,36], and executive dysfunction may play a role in this relationship. Studies have also linked poorer executive control to altered eating and sedentary behaviors [37,38], which may further exacerbate the psychological challenges faced by students with food insecurity and adversely affect academic performance. Our study findings provide new insights into potential pathways whereby students with food insecurity may face greater academic challenges due to poorer executive function. Impairments in organization, planning, or working memory, for instance, may lead to greater difficulties in engaging self-regulatory behavior and potentially result in impaired academic performance. More research is needed to understand the mechanistic link between food insecurity, executive function, eating choices, and academic performance among university students.

The neuroimaging analyses reveal two essential findings involving networks that are central to cognitive function. First, we found greater connectivity between the FPN (the primary network for overseeing goals and regulating behavior) and the left middle temporal gyrus (a component of the DMN that contributes to self-referential and unguided thought) [39] for those with food insecurity. Previous research on these networks indicate that they tend to be anti-correlated at rest in a healthy cognitive state [40,41,42]. In this study, the opposite pattern is observed for students with food insecurity, one of poorer anti-synchrony between these networks. Diminished anti-synchrony between the FPN and DMN has been associated with attention-deficit/hyperactivity disorder (ADHD), suggesting abnormal connectivity between these regions plays a role in executive impairment [43]. Hence, heightened connectivity between the FPN and left middle temporal gyrus may be maladaptive and contribute to executive impairment among those with food insecurity. We find preliminary evidence for this, as greater connectivity between the FPN and left middle temporal gyrus correlated with worse scores on the BRIEF Organize subscale in the food insecure group, suggesting it may be a mechanism of executive impairment.

The students with food insecurity also showed less anti-synchrony between the SN and the precuneus and right middle frontal gyrus compared to the food-secure students. Given that the precuneus is a major hub of the default-mode network [44], these findings suggest a similar pattern of poorer anti-synchrony between key cognitive networks for those with food insecurity. We also found that connectivity between the salience network and the right middle frontal gyrus was significantly correlated with BRIEF Inhibit scores for those with food insecurity, such that greater connectivity was associated with poorer executive function. Previous research has demonstrated that a hub of the FPN lies within the middle frontal gyrus [45]. This further supports the possibility that poor anti-synchrony between key cognitive networks is a mechanism underlying executive dysfunction in students with food insecurity.

This study has several limitations which should be considered. Firstly, the assessment of executive function was based on a self-report questionnaire rather than being objectively measured through standardized assessment. Secondly, this study had a relatively small sample size. We found significant results for our main effects of interest, but exploratory analyses on executive function subscales and correlations between executive function and functional connectivity should be considered exploratory and in need of replication. The pandemic may have impacted students’ perceptions and experiences of food insecurity. Although these findings are potentially relevant to college students with food insecurity, different neural patterns may exist for other populations affected by food insecurity, and future studies should incorporate a broader demographic. Finally, given the study was cross-sectional, inferences cannot be made about how food insecurity and cognitive function are causally related.

In spite of these limitations, this study has several strengths. This cross-sectional study presents novel findings of functional neural differences that may account for poorer executive function in college students with food insecurity. Despite several studies demonstrating a negative association between food insecurity and cognitive function across a lifespan [11,12], no studies have previously studied how functional connectivity differs for those affected by food insecurity. This study also addresses a gap in our understanding of the relationship between executive function and food insecurity in college students. Future research is needed to gain a better understanding of precisely how executive function is affected for those with food insecurity and should directly investigate whether food insecurity moderates the relationship between brain connectivity and executive function. Studies should incorporate larger cohorts and objective cognitive evaluations and should longitudinally investigate whether improving access to nutritious food leads to improvements in neurocognitive health for affected populations.

## 5. Conclusions

Food insecurity persists as a public health problem, including for emerging adults in college. Disrupted modulation of intrinsic brain networks were evident among college students experiencing food insecurity. Specifically, greater functional connectivity between the FPN, DMN, and SN at rest may underpin executive function difficulties for college students with FI, for which the short and long-term effects remain elusive. Future research is needed to understand if differences in brain function among food insecure college students is related to students’ eating behaviors, academic and health outcomes.

## Figures and Tables

**Table 1:** Differences in adverse childhood experiences (ACEs) and race/ethnicity among college students with and without food insecurity (*n* = 40).

	Food Insecure (*n* = 20)	Food Secure (*n* = 20)	*p*-Value
ACEs (*Mean ± SE*)	3.7 ± 0.6	1.6 ± 0.3	0.006
Pell grant status % (*n*)	33.3 (10)	66.7 (5)	0.102
Race/Ethnicity % (*n*)			
White	47.1 (8)	52.9 (9)	0.749
Other	57.5 (12)	47.8 (11)	

**Table 2:** Multivariable linear regression models of the association between food insecurity (FI) and executive function ^1^, adjusting for adverse childhood experiences (ACEs) among study participants (*n* = 40).

Dependent Variable	Predictor	β	Cohen’s D	95% CI
BRIEF Global Executive	FI	8.36 *	0.43	2.32, 14.40
	ACEs	1.69 *	0.42	0.46, 2.93
BRIEF Inhibit	FI	8.25 *	0.45	2.58, 13.9
	ACEs	0.63	0.19	−0.23, 1.78
BRIEF Shift	FI	5.58	0.25	−1.26, 12.62
	ACEs	1.70 *	0.37	0.28, 3.11
BRIEF Emotional Control	FI	3.01	0.14	−3.71, 9.75
	ACEs	1.45 *	0.33	0.07, 2.83
BRIEF Self-Monitor	FI	5.37	0.27	−0.69, 11.43
	ACEs	1.91 *	0.48	0.67, 3.15
BRIEF Initiate	FI	7.64 *	0.32	0.23, 15.1
	ACEs	1.52 *	0.32	0.06, 3.03
BRIEF Working Memory	FI	9.31 *	0.45	2.90, 15.71
	ACEs	2.13 *	0.5	0.82, 3.44
BRIEF Plan	FI	8.02 *	0.34	0.64, 15.39
	ACEs	0.83	0.17	−0.67, 2.33
BRIEF Task	FI	0.79	0.03	−6.76, 8.34
	ACEs	1.91 *	0.38	0.37, 3.47
BRIEF Organize	FI	11.13 *	0.48	3.96, 18.29
	ACEs	0.51	0.11	−0.94, 1.97

Each of the ten multivariable regression models included both FI and ACEs as predictors. FI: Food Insecurity; BRIEF: Behavior Rating Inventory of Executive Function; ACEs: Adverse Childhood Experiences. ^1^ Higher scores indicate poorer cognitive function; all participants were matched by sex and age. * Statistically significant: *p* < 0.05.

**Table 3:** Clusters showing group connectivity differences involving the frontoparietal network (FPN) or salience network (SN).

Component	Cluster (x, y, z)	Region	Size	Cluster p-FWE
FPN	−50, −62, +04	Left middle temporal gyrus	193	0.029429
SN	+16, −50, +30	Precuneus	280	0.000866
SN	+38, +24, +46	Right middle frontal gyrus	169	0.015856

FWE: Family-wise error.

**Table 4:** Exploratory Pearson correlations between BRIEF scores and connectivity values among students with very low food insecurity (*n* = 20) and students with high food security (*n* = 20).

BRIEF Subscale (T-Scores)	Frontoparietal Network (FPN)-Left Middle Temporal Gyrus		Salience Network (SN)-Precuneus		Salience Network (SN)-Right Middle Fontal Gyrus	
	Food Insecure	Food Secure	Food Insecure	Food Secure	Food Insecure	Food Secure
Total (Global Executive)	r = 0.142;	r = −0.315;	r = 0.177;	r = −0.277;	r = −0.090;	r = 0.179;
	*p* = 0.550	*p* = 0.176	*p* = 0.456	*p* = 0.237	*p* = 0.707	*p* = 0.449
Inhibit	r = 0.307;	r = −0.258;	r = 0.402;	r = −0.345;	r = 0.466;	r = 0.066;
	*p* = 0.188	*p* = 0.273	*p* = 0.079	*p* = 0.137	*p* = 0.038 *	*p* = 0.783
Initiate	r = −0.067;	r = −0.282;	r = 0.148;	r = −0.414;	r = −0.147;	r = 0.044;
	*p* = 0.780	*p* = 0.228	*p* = 0.535	*p* = 0.070	*p* = 0.535	*p* = 0.854
Working Memory	r = 0.027;	r = −0.280;	r = −0.029;	r = −0.417;	r = −0.081;	r = 0.113;
	*p* = 0.910	*p* = 0.232	*p* = 0.904	*p* = 0.067	*p* = 0.734	*p* = 0.636
Plan	r = 0.108;	r = −0.424;	r = 0.387;	r = −0.249;	r = 0.052;	r = 0.009;
	*p* = 0.650	*p* = 0.063	*p* = 0.092	*p* = 0.290	*p* = 0.827	*p* = 0.969
Organize	r = 0.503;	r = 0.017;	r = 0.331;	r = −0.146;	r = −0.161;	r = 0.119;
	*p* = 0.024 *	*p* = 0.944	*p* = 0.154	*p* = 0.539	*p* = 0.498	*p* = 0.619

* Statistically significant: *p* < 0.05.

## Data Availability

Deidentified data will be available upon request.
